# Lithium improved behavioral and epileptic symptoms in an adolescent with ring chromosome 20 and bipolar disorder not otherwise specified

**DOI:** 10.1002/ccr3.1796

**Published:** 2018-10-12

**Authors:** Adlane Inal, Boris Chaumette, Maryam Soleimani, Anne‐Marie Guerrot, Alice Goldenberg, Axel Lebas, Priscille Gerardin, Vladimir Ferrafiat

**Affiliations:** ^1^ URHEA Child and Adolescent Psychiatry Intensive Care Unit Centre Hospitalier du Rouvray Sottevile les Rouen France; ^2^ Department of Neurology and Neurosurgery McGill University Montreal Canada; ^3^ Department of Clinical Genetics Rouen University Hospital Rouen France; ^4^ Department of Child and Adolescent Psychiatry Rouen University Hospital Rouen France; ^5^ Department of Neurophysiology Rouen University Hospital Rouen France; ^6^ Centre Compétent Maladies Rares à Expression Psychiatrique Rouen University Hospital Rouen France

**Keywords:** channelopathy, *KCNQ2*, psychosis, seizures, therapy

## Abstract

We present a case of ring chromosome 20 syndrome in a twelve‐year‐old girl, with resistant epileptic disease and severe behavioral impairment that both drastically improved after a lithium challenge. If replicated, this could support the use of lithium as a safe treatment in the management of this severe phenotype.

## 1 BACKGROUND

Ring chromosome 20 syndrome is a rare chromosomal disorder associated with refractory epilepsy, as well as cognitive and behavioral impairments as impulsivity or aggressiveness. Child psychiatrists and neurologists are challenged by these severe symptoms. Antipsychotics may impact the seizure threshold, with a risk of increasing the number of seizures. Hence, it is complicated to initiate psychiatric medication when behavioral disorders are an expression of resistant epileptic disease. Here, we report the case of a 12‐year‐old girl, diagnosed with ring chromosome 20 syndrome. Clinical features included refractory focal impaired awareness seizures, in spite of antiepileptic polymedication, and major cognitive and behavioral impairments. Cognitive impairments were attention, memory, and executive deficit, and behavioral impairments were aggressiveness, irritability, disinhibition, and hyperactivity. As mood symptoms were fluctuant and could be considered as bipolar disorder not otherwise specified (BP‐NOS), we decided to introduce lithium. This treatment was very efficient with an improvement not only in behavioral symptoms but also in epilepsy. Then, we discuss the link between lithium treatment and possible pathophysiology in ring chromosome 20 syndrome. We suggest a possible effect of lithium on *KCNQ2* gene that has been associated with some forms of epileptic disease as well as bipolar disorder.

Ring chromosome 20 syndrome (R(20)), is a rare disorder with a prevalence estimated at around 1 in 30 000‐60 000 births. The mechanism underlying chromosome ring formation is still not clearly understood but it seems to result from rare intrachromosomal fusions. In the majority of cases, the chromosomal aberration is present in only a proportion of cells (mosaicism). Cases with R(20) without mosaicism have also been described.^1^ Most cases are sporadic, and neither gender nor ethnic specificity has been reported.^2^

Phenotypic characteristics include:

- Epilepsy with variable age of onset of seizures, depending on the proportion of cells with R(20), but which typically starts during childhood (1‐17 years old).^3^ Seizures are often refractory to different treatment options including drugs, neurosurgery, and neuromodulation. They are often focal with impaired awareness, occurring during both wake and sleep periods.^4^ Visual ictal hallucinations have been described.^5^
- Cognitive and behavioral impairments which include possible learning disabilities and attention deficit.^6^ Behavioral impairments range from moderate to severe. It seems that poor control of seizures is involved in both increased cognitive and behavioral impairments.^2^
- Dysmorphism, which may be subtle or even absent. Dysmorphic features described in the literature include microcephaly, dental malocclusions, micrognathia, cauliflower‐shaped ears and coarse facial features with slanting eyelids.^7^ Occasional renal and cardiac abnormalities are also reported in this syndrome.^2^

A diagnosis of R(20) could be suspected on presentation of clinical features or could be an incidental discovery. Electroencephalography may be helpful but is generally not very specific. However, abnormal electroclinical patterns characterized by long bursts or trains of rhythmic theta waves, and generalized spike waves associated with seizures (from probable frontotemporal location) have been reported in R(20).^4^

Diagnosis can be confirmed by cytogenetic analysis: karyotype or fluorescence in situ hybridization (FISH) requiring 50‐100 blood cells to exclude mosaicism.^8^

There are few reports on the psychiatric symptoms exhibited in patients with R(20), and no therapeutics are recommended to manage them. We report a case of a 12‐year‐old girl diagnosed with R(20). We describe her psychiatric presentation, which justified the introduction of lithium. Lithium drastically improved both her psychiatric and epileptic symptoms.

Finally, we discuss the possible role of *KCNQ2* gene in the clinical presentation of R(20) and its modulation by lithium, based on the current knowledge about the pathophysiology of epilepsy and bipolar disorder.

## 2 CASE PRESENTATION

The patient was a 12‐year‐old female for whom a diagnosis of R(20) was made in a context of refractory epilepsy with learning disorders.

The diagnosis was confirmed by karyotype at the Department of Clinical Genetics of Rouen University Hospital, Rouen, France, when she was 9 years old. Breakpoints (p13 and q13.3) were confirmed by FISH analysis. Overall, 20% of analyzed cells were found to be R(20). No deletion was found in the subtelomeric regions of chromosome 20 for both short and long arms.

No complications were reported during her mother's pregnancy and delivery. She was eutrophic at birth (weight = 3400 g; height = 50.5 cm; cranial perimeter = 35 cm). APGAR score was 10/10. Regarding psychomotor development, no delays were found during her first years of life.

Learning disorders began in primary school, with reading difficulties. In this context, she was evaluated with standard metric test and prescribed speech rehabilitation. This initial evaluation found attention and memory disabilities with a speech delay. First epileptic seizures appeared at 6 years old.

At the age of 10 years, our patient had a complete neuropsychological evaluation. The Test of Everyday Attention for Children 9 found a deficit in executive function with distractibility, fatigability, and psychological slowness. The Wechsler Intelligence Scale for Children (WISC—IV) was homogeneous without intellectual disability (Verbal Comprehension Index = 79; Perceptual Reasoning Index = 90; Working Memory Index = 88; Processing Speed Index = 96; Full Scale IQ = 83).

Cardiological assessment found a prolapse of the mitral valve without any clinical impact.

At twelve years old, the patient presented a nonconvulsive status epilepticus with altered consciousness and no recovery period for about 1 month. Concomitantly, parents reported an acute change in her behavior. Her antiepileptic treatment was adapted with phenobarbital 60 mg/d, oxcarbazepine 900 mg/d, sodium valproate 1 g/d, hydroxyzine 25 mg/d, and amitriptyline 12 mg/d. EEG showed alternative records switching between normal trace periods and mainly bifrontal slow bursts with spikes and waves and slow rhythmic bursts. This phenotype was associated with major behavioral symptoms including aggressiveness, self‐injury, and sleep disorder. Cerebral MRI found an asymptomatic pineal cyst but no other abnormalities were reported.

Therefore, the patient was hospitalized in our child and adolescent psychiatric unit. Clinical examination found severe psychomotor symptoms including negativism, ambitendency, motor and verbal perseverations, aggressiveness, irritability, and impulsivity. Disinhibition was observed with very foul language, sexual behaviors toward even young peers, and disobedience of rules. Frequent visual hallucinations were reported by the patient and retrospectively confirmed by parents. These hallucinations occurred especially in conditions of low luminosity and were attributed to status epilepticus.^3^

During the first weeks of hospitalization, we observed rapid fluctuations in mood and behavior with oscillations between “depressive‐like” (irritability, aggressiveness, sadness and lethargy) and “manic‐like” symptoms (disinhibition, psychomotor agitation, hyperactivity). The intensity of symptoms was evaluated weekly by a senior physician (VF) using the Aberrant Behavior Checklist (ABC).^10^

Regarding psychiatric features fulfilling the diagnosis criteria for bipolar disorder not otherwise specified (BP‐NOS) (considering the short duration of both depressive and manic symptoms and rapid fluctuations in mood features), and the risk of modifying the epileptic threshold with other therapeutic options as antipsychotics, we decided to challenge this patient with lithium. We introduced lithium 400 mg extended‐release (ER) 2 weeks after her admission. The therapeutic dose (0.8‐1.2 mEq/L) was obtained with lithium 1 g/d ER after 6 weeks.

**Figure 1:**
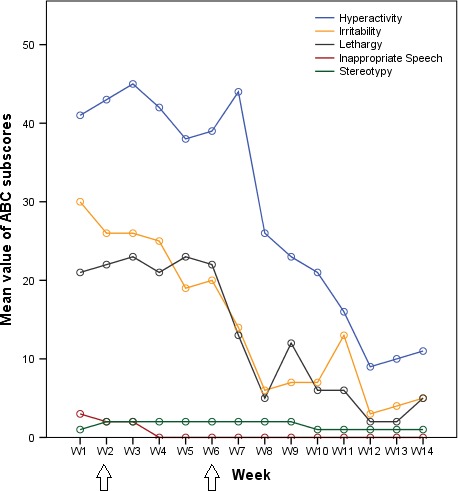
Aberrant Behavior Checklist (ABC) subscores per week (W) show clinical improvement after lithium introduction. Lithium introduced at week 2 (first arrow) and final therapeutic dose reached at week 6 (second arrow)

We observed a drastic clinical improvement especially for impulsivity, irritability, and aggressiveness. ABC subscores were reduced by 30 points for hyperactivity, 25 points for irritability, and 16 points for lethargy between week 1 and week 14 (Figure 1), in line with the classical time frame for action of lithium.

**Figure 2:**
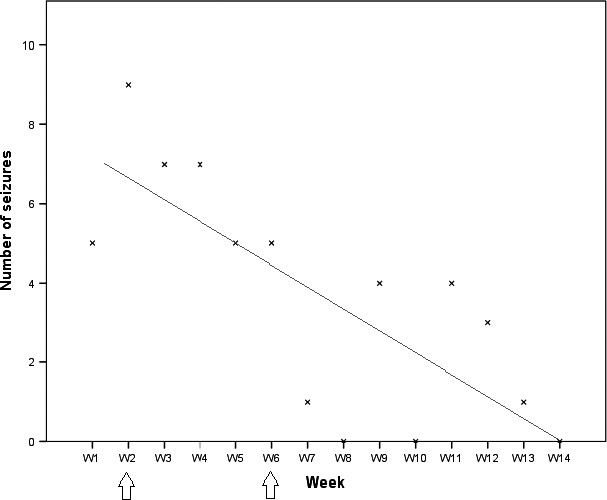
Total number of seizures per week (W). The linear trend curve shows the decrease in the number of seizures. Lithium introduced at week 2 (first arrow) and final therapeutic dose reached at week 6 (second arrow)

Moreover, the average number of seizures per week decreased significantly. Initially, about two to three seizures were observed each day. After 6 weeks, some days were free of seizures, and finally, there was no seizure at all during the last week (Figure 2). However, some oppositional features remained.

Four months after the patient's discharge, monthly follow‐up showed sustained improvement on both epileptic seizure (less than one seizure per week) and mood disorder.

No adverse event concerning lithium was reported, either during hospitalization, or during follow‐up since the end of hospitalization.

This case brings into questions the pathophysiology of neuropsychiatric symptoms in R(20). We found rationale to justify the interest of lithium in R(20) behavioral and epileptic mechanisms.

## 3 DISCUSSION

R(20) is a rare condition with few reports of treatment challenge. The drastic clinical improvement obtained with lithium in our patient is particularly interesting. Indeed, unlike antipsychotics, lithium has no deleterious effect on epileptic threshold, and it has even been reported to be protective against seizures.^11^, ^12^, ^13^ It has shown clear efficacy on impulsivity and aggressiveness in a pediatric population.^14^, ^15^, ^16^ It has also shown its effectiveness in managing pediatric manic episodes and as maintenance strategy for pediatric bipolar disorder.^16^, ^17^ However, efficacy on depressive symptoms of pediatric bipolar disorder seems to be much less clear 16 and further studies should evaluate its use in refractory or recurrent major depression in children with confirmed familial risk of bipolar disorder.^17^ Lithium is largely available worldwide and quite safe compared to other drugs, even in a juvenile population.^16^, ^17^, ^18^ Concomitant reduction in seizure frequency and behavioral symptoms has already been described in other cases of R(20).^6^ The reason for this concomitant improvement is not well understood, but it suggests possible shared pathophysiological mechanisms. One hypothesis is that R(20) pathophysiology is due to the deletion or the silencing of the *KCNQ2* gene leading to potassium channelopathy.^19^ The chromosomal region involved in ring formation is located near the *KCNQ2* gene, which encodes voltage‐gated potassium channels in neurons. *KCNQ2* is involved in many forms of epilepsy ranging from benign to most severe.^20^ Deletion of *KCNQ2* can occur in patients with R(20) due to local rearrangements.^1^ Additionally, several cases have been reported with typical epileptic manifestations of R(20) syndrome without *KCNQ2* deletion, probably due to a dysregulation of its expression or telomeric instability leading to interstitial deletion.^21^ *KCNQ2* has been described to be an epistatic interactor of genes associated with bipolar disorder as *ANK3* 22 or *PPP2R2C*.^23^

Considering the bipolar disorder NOS presentation of our patient and data in the literature on links between epilepsy and bipolar disorder,^24^, ^25^, ^26^ we suggest there may be a link between resistant epileptic disease and bipolar disorder NOS in R(20). Therefore, we extrapolate that bipolar disorder features could be triggered or exacerbated by status epilepticus.

**Figure 3:**
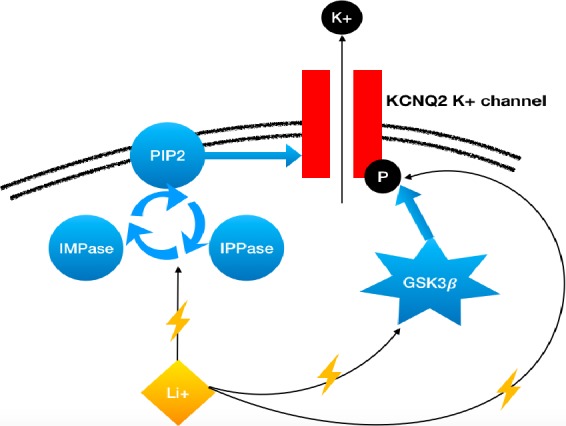
Simplified schematic representation involving supposed mechanisms of action of Lithium (Li+). This drug maintains the K+ channel open and increases neuron hyperpolarization which becomes less responsive to excitatory postsynaptic potential resulting in hypoexcitability of the neuron. PIP2, phosphoinositide biphosphate; IPPase, inositol polyphosphate and phosphatase; IMPase, inositol monophosphate phosphatase; GSK3, glycogen synthase kinase 3 beta

In a previous case report of an 8‐year‐old girl with mosaic R(20), authors reported a remarkable improvement in seizure control with the antiepileptic drug ezogabine.^27^ Unfortunately, ezogabine has been removed from prescription after a warning from the Food and Drug Administration due to retinal pigmentary changes and blue skin discoloration with chronic use.^28^ Interestingly, ezogabine and lithium share similar final pathways and could both trigger the opening of potassium voltage‐gated channels.^23^, ^27^ By maintaining the *KCNQ2* channel open, they may reduce the excitability of the postsynaptic neuron. In 2007, Borsotto et al^23^ extensively described how lithium might impact *KCNQ2* by two mechanisms (Figure 3). First, lithium inhibits GSK3β, an enzyme that is involved in the phosphorylation of *KCNQ2*. This phosphorylation closes the K+ channel making the neuron much more excitable. Consequently, the inhibition of this phosphorylation by lithium may reduce the excitability of the cell. Second, lithium is involved in the regulation of second messengers, especially the phosphatidyl inositol pathway. Phosphoinositide biphosphate (PIP2) synthesis is downregulated by enzymes including inositol monophosphate phosphatase (IMPase) and inositol polyphosphate phosphatase (IPPase), which are both inhibited by lithium. PIP2 is another important *KCNQ2*‐regulating actor.

Altogether, these findings suggest that lithium may interact with *KCNQ2* channel functioning and are in line with those of our case report. Moreover, if replicated, lithium could represent an interesting therapeutic option for patients with severe psychiatric manifestations secondary to a complex neurodevelopmental disorder.

## 4 CONCLUSION

To the best of our knowledge, this is the first report of the positive effect of lithium on both behavioral and epileptic symptoms in a case of ring chromosome 20 syndrome. If replicated, this observation could support the use of lithium as a safe treatment in the management of this severe phenotype. Despite our limited knowledge of the molecular mechanisms of R(20) pathophysiology, prescribing lithium also has a rationale likely involving *KCNQ2*. This gene dysregulation and more generally channelopathy are of interest in neuropsychiatric conditions and warrant further exploration to identify new efficient treatments.

## CONFLICT OF INTEREST

None declared.

## AUTHORSHIP

AI: is a child and adolescent psychiatry resident, followed up the patient during her hospitalization in child and adolescent psychiatry unit, collected and interpreted the data, drafted the manuscript, and interpreted the final draft. BC: is a psychiatrist, collected and interpreted the data, drafted the manuscript, interpreted the final draft, and critically revised the manuscript for important intellectual content. MS: is a child and adolescent psychiatrist, followed up the patient during her hospitalization in child and adolescent psychiatry unit, and interpreted the data and final draft. A‐MG: is a clinical geneticien, has suspected the diagnosis of R(20), validated the further genetic explorations, and interpreted the data and final draft. AG: is a clinical geneticien and interpreted the data and final draft. AL: is a neuropaediatrician, has suspected the diagnosis of R(20), currently following up the patient, and interpreted the data and final draft. PG: is a child and adolescent psychiatrist and interpreted the data and final draft. VF: is a child and adolescent psychiatrist, followed up the patient during her hospitalization in child and adolescent psychiatry unit, collected the data, drafted the manuscript, critically revised the manuscript for important intellectual content, and interpreted the data and final draft.
